# Blood-based Genomic Profiling of Circulating Tumor DNA from Patients with Advanced Pancreatic Cancer and its Value to Guide Clinical Treatment

**DOI:** 10.7150/jca.43087

**Published:** 2020-04-27

**Authors:** Hengchao Li, Yang Di, Ji Li, Yongjian Jiang, Hang He, Lie Yao, Jichun Gu, Jiajun Lu, Jia Song, Shiqing Chen, Shangli Cai, Chen Jin, Zhou Yuan, Deliang Fu

**Affiliations:** 1 Department of Pancreatic Surgery, Huashan hospital, Fudan University, Shanghai, 200040, China.; 2 Department of General Surgery, No.6 People's Hospital, Shanghai Jiaotong University, Shanghai, 200233, China.; 3 The Medical Department, 3D Medicines Inc., Shanghai, 201114, China

**Keywords:** Pancreatic cancer, Circulating tumor DNA, Sequencing, Genomic feature, Personalized therapy

## Abstract

**Objective**: Pancreatic cancer (PC) is a malignant tumor with limited therapeutic choices and extremely poor prognosis. Personalized therapy based on gene alternations is a promising choice. Considering tumor heterogeneity, the practice of ctDNA analysis has drawn the attention. Here, we try to assess the applicability of ctDNA in PC.

**Methods and materials**: Next generation sequencing (NGS) was performed from blood samples of 223 PC patients and tissue sample of 564 PC patients. Genomic data from the TCGA database were also utilized. In addition, two cases received personalized treatment based on ctDNA sequencing results were reported.

**Results**: Based on ctDNA sequencing, the genomic features of PC was revealed. Totally, 68.2% of patients detected at least one reportable genomic alteration (GA) from ctDNA. The frequently altered genes were *KRAS* (53.5%), followed by *TP53* (52.8%), and *CDKN2A* (15.1%). Cell cycle control (8%) and DNA damage response (8%) pathways enriched the most mutated genes. Compared with mutations from tissue samples and a tissue-genomic database, similar frequencies of GAs were detected from ctDNA. The first two highest frequent mutation of genes were the same, but some of mutated genes were inclined to be observed in ctDNA, like *AR*. And two cases who received personalized therapy achieved better clinical benefit.

**Conclusion**: Blood-source ctDNA sequencing could be regarded as a meaningful complement to tissue testing, and might guide clinically therapeutic regimen.

## Introduction

Pancreatic cancer (PC) is one of the most leading lethal malignant tumors, with highly poor prognosis. Surgery is the uniquely curative option, however, less than 20% of newly diagnosed patients are eligible to resection. What's worse, even these patients received curative resection, up to 85% of patients occurs recurrence or metastasis 1. Chemotherapy, like the single-agent gemcitabine, is the treatment cornerstone for advanced PC patients 2. And these days, combination chemotherapy regimens, such as gemcitabine plus nab-paclitaxel 3, have further improved the clinical response. But considering the tumor biology, the phenomenon of resistance to chemotherapy is still unsolved 4.

Target therapy and immune checkpoint inhibitors (ICIs) are the promising therapies in several malignant tumors. But for PC, most researches using small molecule inhibitors or monoclonal antibodies did not screen patients' genomic information, resulting in a lack of clinical benefit 5, 6. Some studies indicated that patients who harboring special biomarkers, like microsatellite instability-high (MSI-H), could more easily obtain clinical benefit from ICIs than the others 7. Hence, screening PC patients for personalized therapy is one of the ways to improve treatment benefit of target therapy or ICIs. Unfortunately, most of the PC patients are initially diagnosed as advanced stage and obtaining the tissue for genetic sequencing is difficult. Thus, liquid biopsy has been the researching focus and may become an alternative way.

Currently, several studies have been launched in liquid biopsy field of PC, especially circulating tumor DNA (ctDNA). Sausen et al indicated that ctDNA could be used as the diagnostic biomarker with a specificity of more than 99% 8. Several papers have also confirmed that ctDNA is an independent prognostic factor 9, 10. However, the research on using ctDNA to reveal tumor heterogeneity and guide clinical decision making are relative limited.

Herein, to assess the applicability of ctDNA, we compared the sequencing results among ctDNA from blood sample, tissue DNA (tDNA) from tissue sample, and DNA from TCGA database. In addition, the genomic landscape from ctDNA was further analyzed. To confirm the clinical meaning of ctDNA sequencing, two cases who received personalized treatment based on the sequencing results were also reported.

## Materials and Methods

### Sample collection and clinicopathologic data

From January 2017 to June 2019, blood or tissue samples from patients with primarily diagnosed as advanced stage PC were collected in Huashan hospital, Fudan University. Genomic profiling of ctDNA or tDNA was tested in a CLIA-accredited/CAP-certified laboratory (3D Medicines Inc., Shanghai, China). A waiver of informed consent form was signed by each patient, and the study was approved by the ethics committee of the hospital. The clinicopathologic characteristics including age and sex were collected.

### DNA extraction, sequencing and data analysis

The assay methodology of DNA extraction, sequencing and analysis followed the methods described in published paper 11. Next-generation sequencing (NGS) targeted 150 cancer-related genes were performed on the NextSeq500 platform (Illumina, CA, USA). The average coverage depth after de-duplicating reads was 3000× for ctDNA with unique identifiers, and 500× for tumor specimens. Sequencing results were analyzed for somatic genomic alterations (GAs) at low mutation allele frequency (AF) which included single nucleotide variant (SNV), copy number variation (CNV) and fusion. Maximum somatic AF (MSAF) was defined as the maximum AF (0.1% < AF < 35%) of all the somatic alterations identified per sample. Single nucleotide polymorphism (SNP) was removed from the calculations of MSAF, but variants of unknown significance (VOUS) were kept. And VOUS were excluded in the other analyses of the whole study. Clinically relevant GAs were named as alternations which could be targeted by currently available or under-researching anti-cancer drugs. Data from TCGA from cBioPortal was extracted in January 2019 12, 13.

### Statistical analysis

The demographic characteristics of patients were compared via the Chi-Square (χ^2^) test or T test. All P-values presented were two-sided, and associations were considered significant if the P-value was less than 0.05. Statistical analyses were performed using the SPSS statistical package, version 20.0 (SPSS Inc®, Chicago, Illinois, USA).

### Case presentation

**Figure 1:**
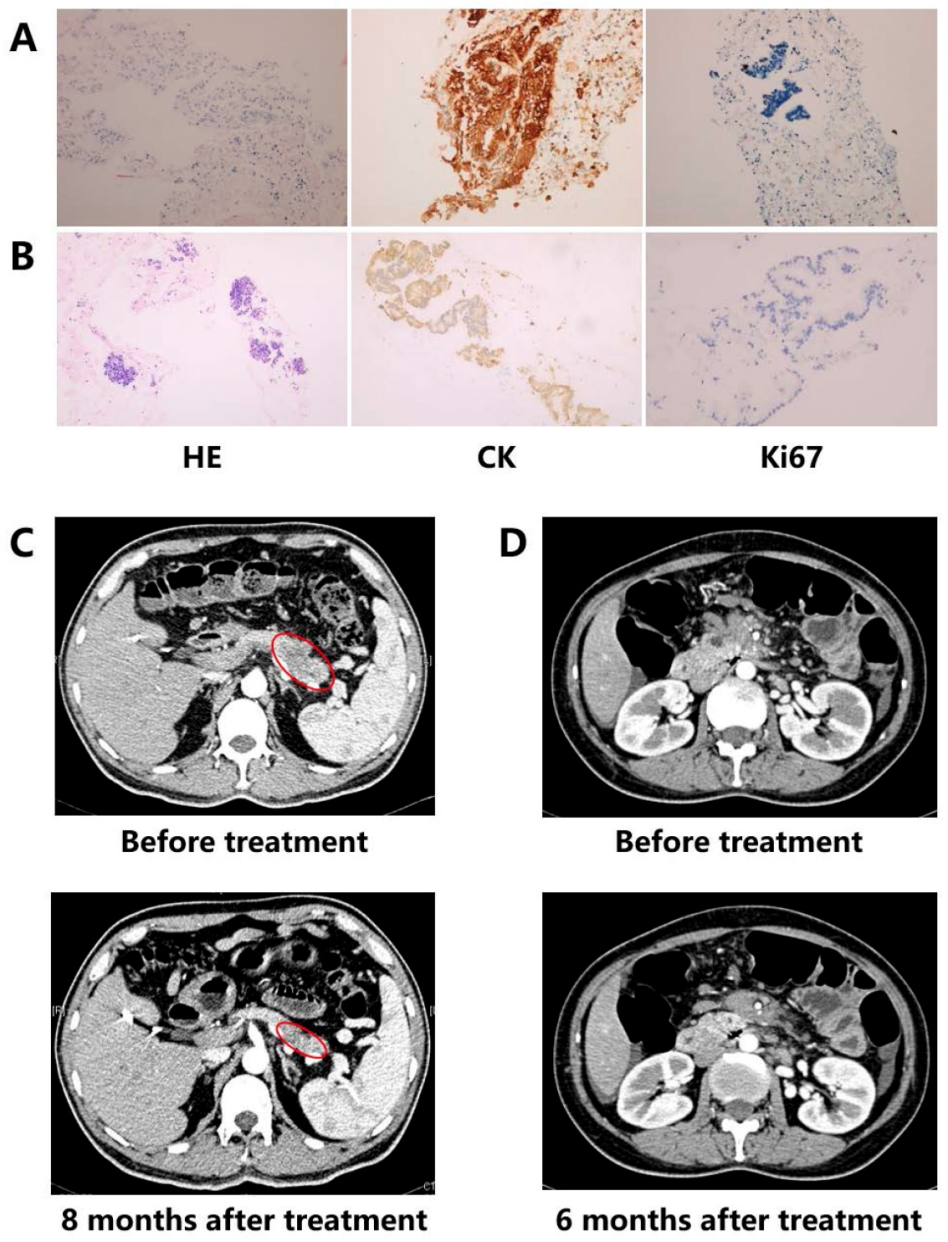
** Detection results of immunohistochemical (IHC) analysis and imaging.** (A) Case 1: IHC results (x100); (B) Case 2: IHC results (x100); (C) Case 1: imaging results; (D) Case 2: imaging results.

Two cases benefited from personalized treatment based on NGS of ctDNA were presented. Patient 1 was diagnosed as a poor differentiated pancreatic ductal adenocarcinoma (PDAC) (stage IV) (Figure 1A). Considering the actually poor health and personal wishes, conventional chemotherapy was not allowed. DNA extracted from the blood sample was utilized to discover optional treatment option. Patient 2 was a 46-year old female PC with stage IV (Figure 1B). Although the patient had received one cycle of three chemotherapeutic drugs (fluorouracil, oxaliplatin and gemcitabine), as the severe gastrointestinal reactions, the patient had to quit chemotherapy and provide blood sample to discover additional therapy.

### Data availability

The raw data that support the findings of this study are available from the corresponding author upon reasonable request.

## Results

### Patient characteristics

The basic characteristics of included patients are shown in Table 1. A total of 787 PC patients have undergone next generation sequencing (NGS), included 223 patients in ctDNA cohort and 564 in tDNA cohort. Most of the patients were PDAC. Median age was 63 years (range, 30-85) and 60 years (range 27-85) in ctDNA cohort and tDNA cohort, respectively. 57.0% of patients in ctDNA cohort and 60.6% of patients in tDNA cohort were men.

As shown, ctDNA detected rate was a little lower in the older patients than the young (P=0.001), however, sex was not associated with ctDNA detection (P=0.157).

### Sequencing results and functional spectra in ctDNA cohort

**Figure 2:**
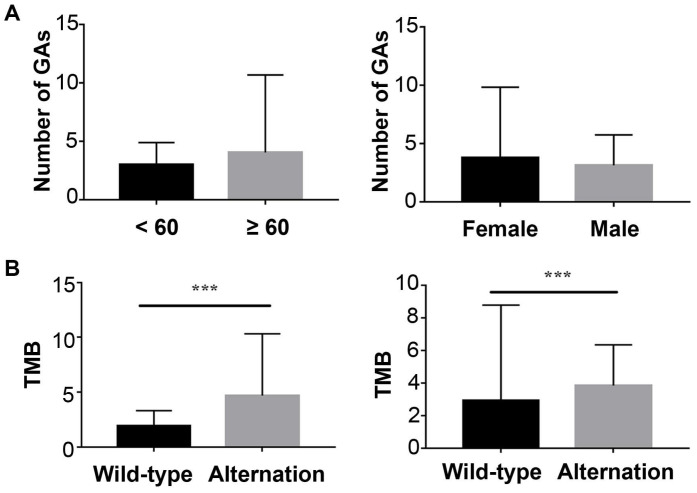
** The Correlation of ctDNA sequencing results and baseline characteristics.** (A) left: Correlation of genomic alternations and age; right: Correlation of genomic alternations and sex; (B) left: Relationship between tumor mutation load and *KRAS*; right: Relationship between tumor mutation load and *TP53*.

In ctDNA cohort, 152 (68.2%) had detected ctDNA with that the maximum somatic allele frequency (MSAF) was greater than zero, and among ctDNA-positive patients, the median MSAF was 1.25%. Each case averagely identified 3.4 genomic alterations (GAs). The number of GAs was not associated with sex or age (Figure 2A).

**Figure 3:**
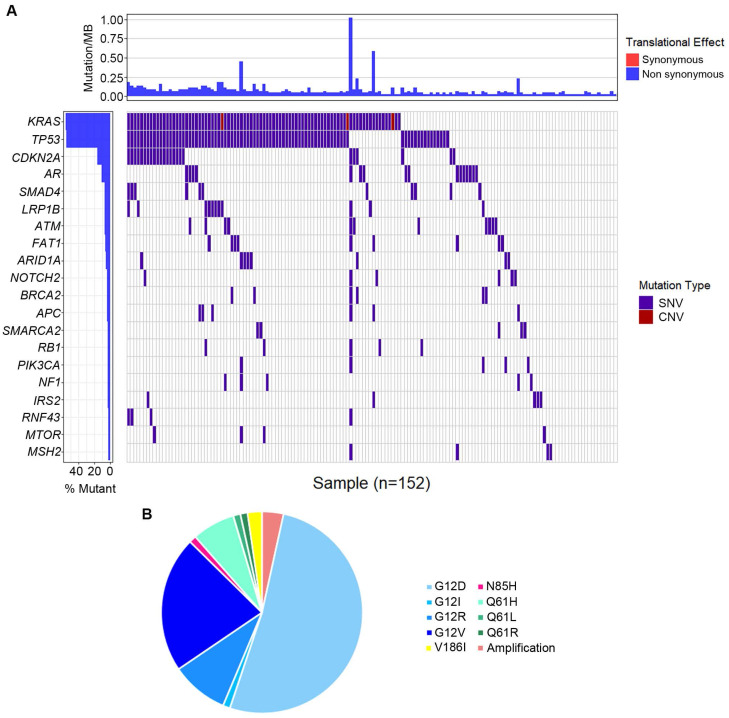
** Genomic alternations detected in circulating tumor DNA (ctDNA).** (A) The whole mutation landscape; (B) Summary of *KRAS* mutations.

The most frequently altered genes were *KRAS* (53.5%), followed by *TP53* (52.8%), and *CDKN2A* (15.1%) (Figure 3A). Several potential drug targets were detected from ctDNA, like *NTRK* family genes (target of FDA-approved Larotrectinib, 3.1%) and DNA damage response related genes *BRCA1* and* BRCA2* (target of olaparib, 5.0%). Among patients with KRAS mutations, 87.0% of patients presented *KRAS* G12 mutation which consisted of G12D (53.6%), G12I (1.2%), G12R (9.5%) and G12V (22.6%), followed by Q61H/L/R, V186I, and N85H (Figure 3B). Besides, we analyzed the association between tumor mutational burden (TMB) and two specific genes (*KRAS* and *TP53*). Both of KRAS alterations and TP53 alterations were associated with higher TMB (P<0.05, Figure 2B).

**Figure 4:**
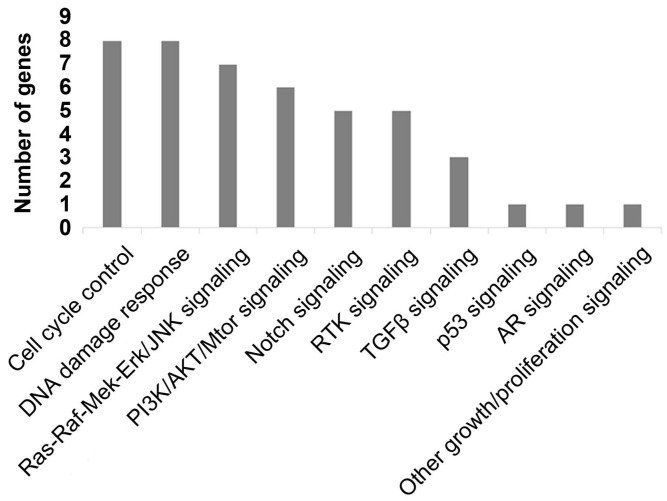
Mapping pathways by frequently mutated ctDNA.

To better comprehend the carcinogenesis in PC, we further analyzed the pathways of the frequently detected genes (Figure 4). In total, ten pathways were mapped, including cell cycle control (8%), DNA damage response (8%) pathways enriched the most mutated genes, Ras-Raf-Mek-Erk/JNK signaling pathway (7%), and PI3K-AKT-mTOR signaling pathway (6%).

### Comparison of ctDNA and tDNA

**Figure 5:**
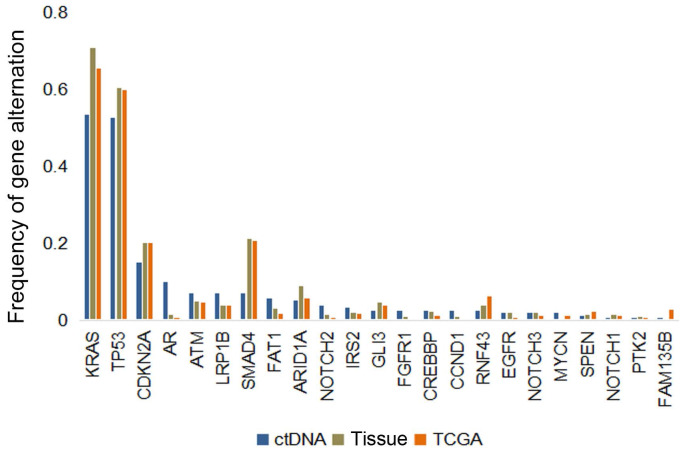
Genomic alterations in circulating tumor DNA (ctDNA) from patients with pancreatic cancer versus in tissue DNA from clinical sample or TCGA database.

The frequencies of common mutated genes in ctDNA cohort were similar with those detected in tDNA cohort and TCGA database (Figure 5). *KRAS* (53.5%, 70.8% and 65.4%, respectively) and *TP53* (52.8%, 60.4% and 59.8%, respectively) were highest frequent mutated genes in these three datasets. However, unexpectedly, some of mutated genes were inclined to be observed in ctDNA cohort, such as *AR*.

### Case management based on ctDNA sequencing

**Patient 1:** A *MLH1* mutation (c.454-1G>A, Table 2), which might result in abnormal mRNA splicing and has been identified as pathogenic mutation, was detected by ctDNA sequencing in patient 1 with PC (Figure 1A). *MLH1* is one of the mismatch repair genes and the deficient mismatch repair is the biomarker of pembrolizumab in solid tumors. Combining the relative lower response of ICI monotherapy in PC, the patient finally received pembrolizumab plus nab-paclitaxel regimen in August 2017. After four medication cycles, the patient experienced rapid clinical symptom relief. What' more, CT scan showed a significant reduction in the pancreatic lesion, and the patient was assessed as a partial response (PR) based on the RECIST guideline (version 1.1, Figure 1C). The serum CA-199 and CA-125 level presented a decline of 92% and 84%, respectively, and both became normal. During the treatment period, there were no treatment-related adverse events. At the time of this writing, the patient was still alive with stable disease (SD) and the progression-free survival (PFS) was more than 24 months.

**Patient 2:** ctDNA sequencing showed patient 2 carried a *BRCA1* mutation (p.R1443*, Table 2) which has been proven as the pathogenicity (Figure 1B). Although poly (ADP ribose) polymerase inhibitor (PARPi) has not approved by FDA in PC, the sensitivity of cells with* BRCA* mutation to PARPi indicated PARPi is one of the available therapies. Then the patient received olaparib from July 2018. After six-month treatment, the patient was evaluated as SD (Figure 1D). The serum CA-199 declined more than 2 fold, and CA125 also presented significantly decreasing (122.7 U/ml to 41.68 U/ml). Although anemia was observed during the period of treating with olaparib, no dose reduction and discontinuation happened. Until the last follow-up, the patient kept SD for nearly 13 months.

## Discussion

Herein, we reported ctDNA mutational landscape of PC patients, analyzed the biological function of mutated genes, probed the concordance between blood and tissue, and validated the clinical application value of ctDNA. These results help us better understand the ctDNA profiling of PC patients.

ctDNA somatic mutation could be detected in nearly 70% of patients. The result was basically consistent with other publications. Pietrasz et al reported that 64.7% of patients with metastatic PC harbored somatic mutations 10, and the proportion was 54.5% in another study 14. Although a higher ctDNA detection rate in advanced PC was founded (nearly reaching 90%) 15. Considering different methodology and diverse quantity of DNA, the discrepancy of DNA detection rate could be accepted. We discovered that patients harboring *KRAS* or *TP53* alternation presented relative higher TMB than that with wild-type. In non-small cell lung cancer, a previous study has revealed that median TMB was significantly higher in the *KRAS*-mutant patients than in the *KRAS*-wild patients (P=0.041) 16. In another study, patients harboring *TP53* mutation showed higher TMB than that with wild-type in lung carcinoma 17.

*KRAS*, *TP53*, *SMAD4*, and *CDKN2A* were the commonly mutated genes in PC tissue that had been revealed by several publications 18, 19. However, data regarding ctDNA profiling is relative limited. Several studies explored ctDNA mutations in PC patients, but sequencing panels with little gene number or PCR-based detected methods were utilized, resulting in limited understanding about the whole landscape 20, 21. In current work, we revealed the ctDNA mutation profiling by 150-genes panel. Our result showed besides *KRAS* and *TP53*, *AR*, *ATM* and *LRP1B* were also frequently mutated. AR, a member of the nuclear receptor superfamily, encodes a ligand-dependent transcription factor. Previous research have extensively reported that AR may play a key role in pancreatic carcinogenesis and cancer development 22. AR signaling is a definitely therapeutic target, especially for hormone-related cancer, such as prostate cancer 23. And in PC, a number of preliminary results on targeting AR signaling have also been reported. A randomized controlled trial reported that the patients receiving flutamide (a non-steroidal antiandrogen) presented significantly longer median survival than the patients with placebo treatment (226 days vs 120 days, P=0.01) 24. A Phase I study showed that treating with enzalutamide combined with chemotherapy represented clinical benefit, with median OS of 9.73 months and PFS of 7.53 months, respectively 25. All these results demonstrated that targeting the AR signaling may be an effective treatment for PC. *ATM* gene encodes a serine/threonine kinase and plays a key role in DNA damage response 26. Previous research have published that ATM loss frequently occurred in the early stage of PDAC and regulated the TGF-β signaling to promote tumor progression 18, 27. In addition, PARPi showed antitumor effect in *ATM*-mutant PDAC in vitro research 28. What's more, olaparib has been approved by FDA to treat PC patients harboring germline BRCA1/2 mutation 29. Thus, the relatively frequent mutation of *ATM* may be regarded as a therapeutic target. *LRP1B* encodes the endocytic LDL-family receptor and is reported as one of the top significantly mutated genes in various malignant tumors 30. Although currently no targeted drugs for *LRP1B* have been developed, several research have revealed the positive association of *LRP1B* mutation and TMB, and *LRP1B* mutation may enrich for ICI responsiveness 31, 32. All these results provide a potentially promising treatment prospects.

PC, widely known as a highly heterogeneous histology, always presented drug resistance and rapid tumor progression. Thus, merely based on DNA mutations from tumor tissue, the disease management may be relative tough. In current paper, we observed the mild consistence between ctDNA and DNA of tissue (both clinical tissue and data from the TCGA database). Although the top frequently mutated genes were similar, some of the gene alternations were respectively detected from blood and tissue with different frequency. Waddell et al have revealed the mutational landscape of PC by whole-genomes sequencing. They indicated that *KRAS*, *TP53*, *CDKN2A*, and *SMAD4* (over than 50%) were the most highly frequent mutated genes detected from the tissue DNA 18. Detecting the genic mutations of the ctDNA, several diverse sequenced results were mentioned. Namely, merely 9.6% of the patients detected* SMAD4* mutation 10. The different sequencing method, different stage might be the main reason. Currently, no previous work have directly compared the difference mutations between ctDNA and tissue DNA. And what's more, the sequencing results in diverse human species have also not been compared. These gaps need more ctDNA sequencing studies to make up.

Although the favorable responses of ICI monotherapy have been seen in solid tumors, it is not a promising strategy for PC. Thus the new treatment concepts emerge as the time require. Firstly, the immunotherapy-based combination regimens should be considered. Previous research have proved that chemotherapy was associated with apoptosis by increasing tumor antigen presentation and leading to the depletion of regulatory T cells 33, 34. A phase Ib/II trial published an ORR of 25% in PDAC patients who received pembrolizumab plus chemotherapy 35. An interim result from a phase I study indicated that the combination of pembrolizumab and nab-paclitaxel achieved an ORR of 18% and additionally added gemcitabine rising ORR up to 50% 36. Probing the predictive factors of ICIs' response is another choice. dMMR/MSI-H has been approved by FDA to screen patients benefiting from immunotherapy 7. To summarize, the patient of case 1 received ICI plus chemotherapy as *MLH* mutation was detected. For case 2, a distinct case who received target therapy based on detected gene mutation was shown. The FDA granted orphan drug designation to PARPi olaparib for treating patients with PC on October, 2018. What's more, maintenance olaparib was proven curative to the treatment of BRCA germline mutated pancreatic cancer 29. All these results supported that the patient accepted olaparib monotherapy in advance, and luckily, the patient presented clinical response. We find a common ground from these two cases. Namely, the material receiving NGS was ctDNA from the blood. And each of the patients was treated with personalized therapy based on the detecting results. The obvious clinical benefit was observed, and these cases could provide some reference for clinical management of PC patients, especially personalized therapy.

One limitation of this paper is failure to collect the whole baseline clinical characteristic and therapeutic regimens. Many clinical features may affect the detection of ctDNA that have been announced 11, 37. In the future, probing the association between clinical information and molecular information is urgently needed.

In conclusion, the current work revealed the ctDNA mutational landscape and directly compared the consistency of gene mutations from ctDNA and tissue DNA. Our results indicated that the utility of ctDNA testing in PC was an importantly complementary approach to the tissue sequencing. These results help us better understand the ctDNA profiling of PC patients, and may provide more information for PC' personalized therapy, which need more studies, especially large prospective clinical studies.

## Figures and Tables

**Table 1:** Characteristics of pancreatic cancer patients with ctDNA samples or tumor tissue samples.

Characteristic	ctDNA samples	Tissue samples
Cases	223	564
Median age, year (range)	63 (30-85)	60 (27-85)
Sex (male vs female)	127 vs 96	342 vs 222
Subtype (ductal adenocarcinoma vs other)	200 vs 23	479 vs 85
MSAF>0, n (%)	152 (68.2%)	551 (98.4%)
Median MSAF	1.25%	19.6%
Average GA/case	3.4	4.6

Note: MSAF: maximum somatic allele frequency; GA: genomic alternation.

**Table 2:** List of gene alternations from the two patients.

Patient Number	Gene	Mutation type
Patient 1	*MLH1*	c.454-1G>A
Patient 2	*BRCA1*	p.R1443*

## Abbreviations

PC: pancreatic cancer
PDAC: pancreatic ductal adenocarcinoma
ICI: immune checkpoint inhibitor
MSI-H: microsatellite instability-high
TMB: tumor mutational burden
ctDNA: circulating tumor DNA
GA: genomic alteration
AF: allele frequency
SNV: single nucleotide variant
CNV: copy number variation
MSAF: maximum somatic allele frequency
SNP: single nucleotide polymorphism
VOUS: variants of unknown significance
PARPi: poly (ADP ribose) polymerase inhibitor
